# Journal of Experimental & Clinical Assisted Reproduction: shaping the future of research and practice in reproductive endocrinology/infertility

**DOI:** 10.1186/1743-1050-1-1

**Published:** 2004-09-02

**Authors:** E Scott Sills, Robert M Winston, Gianpiero D Palermo

**Affiliations:** 1 Editor-in-Chief, Journal of Experimental and Clinical Assisted Reproduction; 2 Division Director, Reproductive Endocrinology and Infertility, Department of Obstetrics and Gynecology, Atlanta Medical Center, Atlanta, Georgia USA; 3 Director for Oocyte Donation, Georgia Reproductive Specialists LLC, Atlanta, Georgia USA; 4 Senior member, Editorial Board, Journal of Experimental and Clinical Assisted Reproduction; 5 Professor of Fertility Studies, Imperial College, London, United Kingdom; 6 Editor-in-Chief, Journal of Experimental and Clinical Assisted Reproduction; 7 Director of Assisted Fertilization, Cornell Institute of Reproductive Medicine and Infertility and Associate Professor, Weill Medical College of Cornell University, New York, New York USA

**Keywords:** publishing, reproductive medicine, internet, research, trends

## Abstract

*Journal of Experimental & Clinical Assisted Reproduction *is an open access, online, peer-review journal publishing papers on all aspects of research into reproductive endocrinology, infertility, bioethics and the advanced reproductive technologies. The journal reports on important developments impacting the field of human reproductive medicine and surgery. The field exists as a sub-specialty of obstetrics & gynecology, focusing on the diagnosis and treatment of complex human reproductive problems. The continued growth of this relatively new field depends on quality research by proven scientists as well as junior investigators who, together, make contributions to this area of medical and surgical practice. The publishing revolution made possible by internet technology presages a bright future for continued interdisciplinary collaboration among researchers. Against this background, *Journal of Experimental & Clinical Assisted Reproduction *exists for the scientific community to facilitate this scholarly dialogue.

## Introduction

In early 2002, planning for *Journal of Experimental & Clinical Assisted Reproduction *began in Atlanta after discussions with London-based publisher BioMed Central to develop a new electronic peer-reviewed journal reporting on the rapidly growing field of reproductive endocrinology and infertility. Working with colleagues who shared this vision, an exploratory organizational group was established to design a journal that would not only meet the current demands of the specialty, but also anticipate its challenges and needs for the future.

By the time of its formal launch in July 2004, *Journal of Experimental & Clinical Assisted Reproduction *already had received submissions and inquiries from authors seeking to publish manuscripts in the categories of original research, review, case report, opinion/debate, medical history, and letters. Although column space does not constrain electronic publishing in the same way as traditional print journals, our peer-review process determined that not every submission was appropriate for publication. Hence, from the first instance the journal has pledged to observe a peer-review policy that ensures only the best quality manuscripts are published.

Our editorial board membership reflects a depth of expertise required to guide editorial policies of a journal that aspires to join the top tier of selective scientific journals. *Journal of Experimental and Clinical Assisted Reproduction *is fully committed to the philosophy of Open Access as articulated in the following policies:

## Free, full-text access for all articles

While the availability of published articles on the journal's homepage provides high and unrestricted visibility for accepted articles, contents of *Journal of Experimental & Clinical Assisted Reproduction *(ISSN 1743-1050) are also accessioned in PubMed Central, maintained by the U.S. National Library of Medicine – the world's largest medical library. Accordingly, all journal publications are available free and without password requirements to anyone with internet access. Furthermore, the platform provided by our publisher, BioMed Central (NLMID b101153627), allows author(s) to track how frequently their manuscript has been accessed and viewed by the public at no charge.

## Journal scope and article categories

The journal reports on important developments impacting the field of human reproductive medicine and surgery. We accept manuscripts describing research in reproductive endocrinology, infertility, bioethics and the advanced reproductive technologies. At the discretion of the Editors, conference proceedings may be considered for publication by special arrangement.

## Rapid publication

After a manuscript is received via the journal's on-line electronic submission system, selection of referees and editorial review is underway within a few business days. Our peer review process evaluates the appropriateness and suitability of all submitted manuscripts, as well as supplying authors with any additional requirements or modifications needed before the article can be published. We seek to reach an editorial decision on a manuscript within three weeks of its initial receipt, although author delays in addressing referees' comments are likely to extend this timeline. Questions regarding the suitability of a proposed submission may be sent to the journal office at , although this is not a requirement

## Formal peer-review policy

The abstract of each manuscript is reviewed by two internal referees, which may include members of the Editorial Board. Authors can expect a response regarding this preliminary review within one week, and manuscripts judged outside the scope of the journal are identified accordingly. Submissions considered to be appropriate for the journal are reviewed by at least one independent referee. Referees' comments are relayed to corresponding authors in a confidential/anonymous manner, and all communication regarding submissions is through the journal's editorial office. The Editorial Office will review commentaries, together with input from an Editorial Board member with the relevant expertise. It may be necessary to enlist additional reviewers and/or statisticians in certain cases to assure the highest quality manuscripts are chosen for publication.

## Monetary author costs

For the first six months, no author charges will apply to any work accepted for publication in *Journal of Experimental & Clinical Assisted Reproduction*. However, for each manuscript accepted thereafter, our publisher will charge a modest fee comparable to the cost of a single color page charge in a print journal (see ). This fee may be waived in hardship cases or for selected authors without budget support, provided that such a waiver request is made at the time of manuscript submission. These policies assure no publishing bias will exist against residents, fellows, or other investigators with little or no research funding.

## Copyright retained by authors

We do not require authors to assign their copyright claim to the publisher as a condition of publishing any article. This means that our authors keep the copyright to their article with the freedom to include article components in subsequent published work, to submit the article in full to colleagues, or to include the work in their own homepage(s) without the publisher's prior authorization or permission.

## Conclusion

With the beginning of a new century of medical research, library privileges and institutional journal holdings will continue to be eclipsed by electronic publishing and unrestricted access to the internet. The publishing revolution made possible by such technology presages a bright future for continued interdisciplinary collaboration among researchers. Against this background, *Journal of Experimental & Clinical Assisted Reproduction *exists for the scientific community to facilitate this scholarly dialogue.

## Author contributions

ESS, RMW, and GDP drafted and reviewed the manuscript.

